# Gingiva squamous-cell carcinoma in a non-smoking patient with occupational exposure to solvent siphoning using mouth: case report and literature review

**DOI:** 10.3389/fpubh.2024.1370767

**Published:** 2024-05-02

**Authors:** Céline Lamouroux, Louis Brochet, Philippe Zrounba, Barbara Charbotel, Béatrice Fervers

**Affiliations:** ^1^Université de Lyon, Université Lyon 1, Université Gustave Eiffel-IFSTTAR, UMRESTTE, UMR T 9405, Domaine Rockefeller, Lyon, France; ^2^Hospices Civils de Lyon, CRPPE-Lyon, Centre Hospitalier Lyon Sud, Pierre Bénite, France; ^3^Surgical Oncology Department, Centre Léon Bérard, Lyon, France; ^4^Department Prevention Cancer Environment, Centre Léon Bérard, U1296 INSERM Radiation, Defense, Health and Environment, Lyon, France

**Keywords:** case report, oral cavity squamous cell carcinoma, solvent exposure, occupational, screen printer, siphoning

## Abstract

**Background:**

While overall head and neck cancer incidence decreases due to reduced tobacco and alcohol consumption, the incidence of HPV negative oral cavity squamous cell carcinoma (SCC) is raising in several industrialized countries, especially in non-smoking and non-drinking patients.

**Case presentation:**

We document a case of gingiva SCC in a 56 years old never-smoker patient reporting low alcohol consumption and unusual occupational solvent exposure. The HPV-negative lesion was surgically removed in 2018, and the patient remains in complete remission 4 years after recurrent surgery in 2019. In 2021, the patient was referred to the occupational cancer consultation. The patient worked as screen printer for 18 years. He reported mouth siphoning every 2–3 days to transfer organic solvents (mainly aromatic hydrocarbons and ketones) from containers into smaller recipients, with regular passage of solvents into his mouth.

**Conclusion:**

According to the literature, the frequency of solvent siphoning using mouth is likely to be underestimated. While our review did not find studies reporting longterm consequences to the oral cavity of mouth siphoning, current evidence supports a positive association of upper aero digestive tract SCC with occupational exposures to organic solvents and printing processes. In absence of major extraprofessional factors, the HPV-negative gingiva SCC of this patient might be attributable to the regular occupational oral solvent exposure. While the available evidence remains limited to formally establish a causal relationship, clinicians should investigate this hazardous work practice in patients with OSCC and history of solvent exposures.

## Introduction

With almost 355,000 new diagnoses and over 177,000 deaths worldwide in 2018 (1), the oral cavity represents the most frequent location of head and neck Squamous Cell Carcinomas (SCC) of the upper aerodigestive tract, accounting for more than 25% of cases. The gingiva is reported as a relatively rare location, representing less than 10% of oral cavity SCC (OCSCC) (2). In most cases, the development of OCSCC is preceded by dysplasia evolving into carcinoma *in situ*, and further progression into invasive SCC (3).

While overall head and neck cancer incidence decreased due to reduction of tobacco and alcohol consumption, the incidence of OCSCC has been described as increasing in several industrialized countries (4–7). Increasingly, Human Papillomavirus (HPV) infection is recognized as a causal etiologic factor for cancers of the oropharyngeal regions, especially at the base of tongue, the lingual tonsil and soft palate (8). Yet, no consistent associations with HPV (9), herpes simplex virus or Epstein–Barr virus (10) have been shown for OCSCC, including gingiva SCC. To date, the etiology of OCSCC in HPV negative, non-smoking and non-drinking patients remains unknown. Moreover, the role of occupational factors in OCSCC remains insufficiently known (11, 12). We document here a case of gingiva squamous-cell carcinoma in a 56 years old never-smoker patient with unusual occupational exposure to organic solvents as a screen printer. To investigate the hypothesis of a relationship between occupational exposure to organic solvents and OSCC, raised by the case report, we further complemented the case report with a comprehensive literature search and review pertaining to occupational exposures associated with risk of OCSCC, in particular to organic solvents.

## Detailed case description

### Cancer diagnosis and treatment

**Figure 1:**
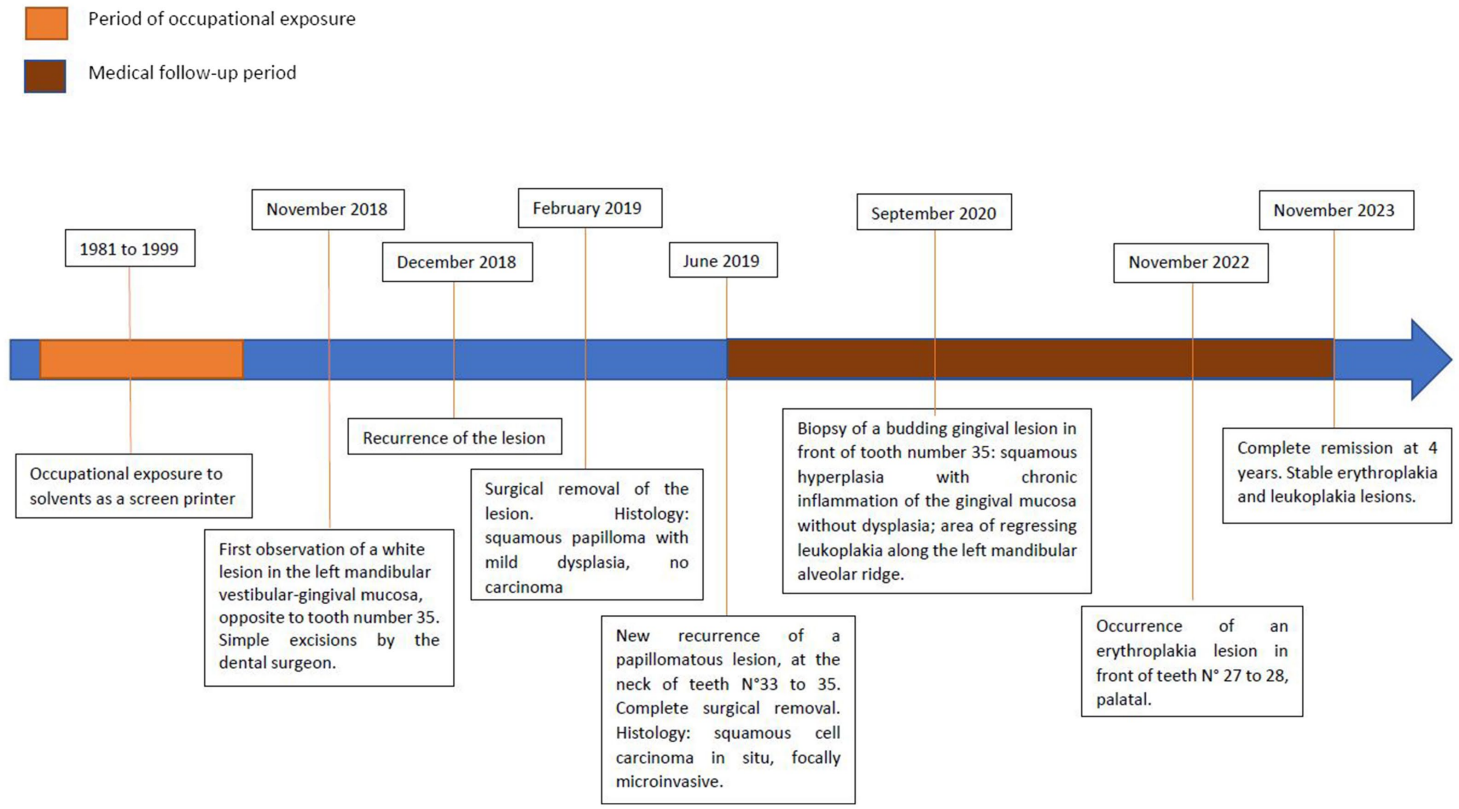
Timeline of the case.

The 56-year-old patient was referred by his dental surgeon to the Leon Berard Comprehensive Cancer Center (CLB), Lyon (France), for recurrence of a white lesion in the left mandibular vestibular-gingival mucosa, opposite to tooth number 35. The dental surgeon had previously performed a simple excision of the lesion in November 2018, without anatomopathological analysis. Figure 1 summarizes the chronological sequence of the case report. Clinically, the lesion had a papillomatous appearance, soft to palpation, elongated, and well delimited, measuring 2.5 cm in the sagittal plane. There was no similar lesion elsewhere in the oral cavity. At time of diagnosis, the patient was otherwise in good health, partially dentate in the maxilla and mandible with good dental health, regularly treated by his dental surgeon. No traumatic tooth or mechanical factor was found.

The recurrent lesion was removed under local anesthesia, in February 2019. Histological examination of the removed specimen found an aspect suggestive of squamous papilloma with mild dysplasia without presence of infiltrating carcinoma.

In June 2019, a novel recurrence of a papillomatous lesion of increased size, developed at the neck of teeth number 33–35, was treated by recurrent surgical removal of the lesion with a mucoperiosteal advancement flap under general anesthesia. Histological examination found a squamous cell carcinoma *in situ*, focally microinvasive, of verrucous architecture, 12 mm long, and associated lesions of mild and high grade dysplasia, and squamous papilloma. The lateral resection boundaries were in healthy or hyperplastic/mild dysplasia mucosa, the deep resection margins were in healthy tissue. For carcinoma, the margins ranged from 2 to 4 mm.

Cervico-thoracic CT scan with contrast injection showed no suspicious cervical lymphnodes, nor mandibular bone lysis in front of teeth number 33–35. The lesion was classified pT1cN0cMx R0 according to the 8th version of the International Union Against Cancer (UICC) (13).

Immunohistochemical staining with anti-p16 antibody was negative. Chromogenic *in situ* hybridization for high-risk HPV DNA with the INFORM III HPV probe (HPV16, 18, 31, 33, 35, 39, 45, 51, 52, 56, 58, and 66) and low risk HPV DNA with the INFORM II HPV probe (HPV 6, 11, 40, 42, 43, 44, 54, 61, 70, 72, and 81) was also negative.

A Multidisciplinary Consultation Meeting recommended active surveillance every 2 months. In September 2020, a biopsy of a budding gingival lesion in front of tooth number 35 showed a focus of squamous hyperplasia with chronic inflammation of the gingival mucosa without dysplasia. The postoperative course was marked by a section of scarred mucosal flange by a Z-plasty. In addition, there was an area of regressing leukoplakia along the left mandibular alveolar ridge not suspicious upon inspection and palpation, and remaining stable during surveillance visits. In November 2022, an erythroplakia lesion appeared in front of teeth number 27 to 28, palatal. In November 2023, the patient remains in complete remission 4 years after the recurrent surgery. The leukoplakia and erythroplakia lesions remain stable. The yearly cervico-thoracic CT scan did not reveal any suspicious lesion.

### Medical history and lifestyle

The patient declared he had never smoked, and reported occasional alcohol consumption on weekends. He had no medical history, no allergies, and took no medication. The patient had no particular family medical history.

### Occupational history and exposures

In December 2021, the patient was referred to the occupational cancer consultation of the CLB (14) to review the job history and workplace exposures. The patient had been working during 18 years, from the ages 18 years to 36 years (1981–1999), as a screen printer in two successive printing enterprises employing two and 150 workers, respectively. His principal tasks consisted in preparing and loading the inks, making the prints, and cleaning the printing machines. The patient reported the manipulation of inks containing lead. Given the employment period, the manipulation of other heavy-metal based pigments (e.g., chromates, cadmium) is likely. Furthermore, he regularly used a solvent mixture for the cleaning of the machines and materials with solvent-soaked rags. The solvent mixture contained aromatic hydrocarbons (e.g., toluene and xylene), and ketones (e.g., cyclohexanone). The presence of benzene in the solvents during the first years of employment is highly probable. To clean the printing screens with solvent-soaked cloths, the patient reported having to lie under the screen, without any personal protective equipment for this task that regularly caused him eye irritation. Also, several times per day, the patient used the same solvents to clean his hands. He took his meals in the atelier. The patient did not present with onychophagia. He described progressive phalangeal hair loss after entering the position, that was partially reversible after having quit the job. The patient further reported that he transferred every 2–3 days, the solvent mixture delivered in large containers, into smaller ones using a tube to prime the siphon by suction. He stated that during this procedure, he regularly got a certain quantity of the solvent mixture in his mouth, frequently a mouthful of solvents. Also, he sometimes accidently swallowed it.

From 1999 to 2004 he worked as workshop manager for the manufacture of road signs in a medium size enterprise with 46 employees. No specific occupational exposures to carcinogens were identified in this position. Since 2004 he works as tramway driver in the public transport of the Metropolis of Lyon, France, exposing him potentially to air pollution.

## Discussion

The originality of the present case report consists in the unusual occupational exposure to solvents in this 56-year-old screen printer presenting with a HPV negative gingiva squamous cell carcinoma. In absence of notable extra-occupational risk factors in this never smoker patient with low alcohol consumption, the regular exposure during siphoning solvents and its potential link with the gingiva SCC warrants further investigation.

Hazardous work practices using mouth suction to siphon transfer substances have been described by very few studies, mainly in vehicle mechanics in older studies or in developing countries to siphon petrol (15–17). In a study among 335 US car equipment mechanics from the 90s, 51% of mechanics reported ever and 11% reported current siphoning petrol by mouth (15). While several case reports (18, 19), reviewed recently by Chen et al. (20) documented over 40 cases of hydrocarbon pneumonitis following workplace or illegal fuel siphonage, we did not identify any study reporting long term consequences to the oral cavity of such practices, including cancer.

A potentially malignant oral lesion, also called dysplasia, is a growth that contains abnormal cells confined to the lining of the oral cavity. It is established that oral potentially malignant lesions and epithelial dysplasia are statistically more likely to progress to cancer, the actual mechanisms are poorly understood, and the prognostic significance of an individual lesion is difficult to determine (21). Consequently, the degree of dysplasia best guides the progression potential of oral lesions. The malignant transformation rate is about 16%, 3–15%, and below 5% for severe, moderate and mild epithelial dysplasia, respectively (22, 23). Clinically, this histopathological lesion can present as leucoplakia, erythroplakia, or papillomatous lesion.

The gingival SCC in the present patient developed through malignant transformation of dysplasia. While being a relatively common benign lesion of the oral cavity, most frequently seen in patients in the 4th and 5th decades, the appearance of squamous cell papilloma on the gingiva is rare (20, 21). Oral cavity squamous cell papilloma has been associated with HPV, with detection rates ranging from 21 to 50% (23, 24). The International Agency for Research on Cancer (IARC) Monographs concluded that, for the oral cavity, there was sufficient evidence for the carcinogenicity of HPV 16, and limited evidence for the carcinogenicity of HPV 18 (25). Yet, the present patient presented with a HPV-negative SCC. Little is known on risk factors for malignant transformation of oral cavity HPV-negative squamous cell papilloma. Dietary and other lifestyle factors, such as physical activity and oral hygiene, are considered relatively minor risk factors (26–28). Of note, outdoor and industrial occupations, as well as exposure to organic solvents have been suggested as risk factor for malignant transformation of sinonasal papillomas (23).

In the absence of established risks factors for OCSCC in this never smoker patient with low alcohol consumption, the unusual and recurrent work-related local exposure to solvents may represent a potential risk factor for dysplasia and SCC. Printing processes are associated with complex pattern of exposure. In the present patient, who worked as screen printer for 18 years, several occupational exposures to established or suspected carcinogens have been identified. These include screen-printing inks containing lead, aromatic amines, chromates, and possibly cadmium, carbon black, cobalt, and formaldehyde (29), as well as organic solvents mainly aromatic hydrocarbons and possibly benzene (30). In addition to the unusual solvent exposure, repeated skin contact occurring in workers using solvents to wash their hands can lead to solvent absorption as well as solvent ingestion through contaminated hands and food (31).

Printing processes, including screen printing, have been classified by IARC as possibly carcinogenic to humans in 1996 with positive associations for lung and bladder cancer (30). The IARC monograph included three case control studies that examined the risk for cancer of the oral cavity and oropharynx in the printing and publishing industry with diverging results (32–34), two studies reporting smoking-adjusted excess risks for cancers of the oral cavity in printing workers (32, 33). A third case–control study on oropharyngeal cancer did not find an increased risk among men employed in the printing industries, whereas a statistically increased albeit not significant risk was found for women (34). Two further studies found a significant excess risk for oral cavity cancers in the printing industry (35, 36). Kvam et al. (35) reported an increased incidence of oral cancers in unskilled workers of the norvegian printing industry [OR 2.12 (95% Confidence Interval (CI) 1.06; 3.79)]. Likewise, Pukkala et al. (36). reported a significantly increased incidence of oral cavity cancers among male printers in the Nordic countries [SIR 1.29 (CI 1.03; 1.59)], but not in women.

Occupational exposures in screen printing processes involve a variety of substances, including various solvents (mainly petroleum solvents) and polycyclic aromatic hydrocarbons (30). An association of oral cavity cancers with exposure to polycyclic aromatic hydrocarbons and solvents has been reported in the literature, but current evidence remains limited (12). Moreover, the IARC has defined exposure to hard and oxidized bitumen during mastic asphalt work or roofing with limited evidence for a causal link with oral cavity cancers (11).

In a previous prospective and systematic assessment of occupational exposures in 154 head and neck SCC cancer patients managed at the Léon Bérard Cancer Center, solvent exposure was observed in 10 out of 67 patients (14.9%) with OCSCC, including three non-smoking patients (14). Several studies have investigated the association between oral cavity cancers and exposure to solvents, yet frequently grouped together with pharyngeal cancers (37). Paget-Bailly et al. (12) in a meta-analysis reported an increased risk for oral cavity SCC, albeit not significant, for occupational exposures to aromatic hydrocarbons, both for high and low exposure levels [meta-RR 1.25 (CI 0.98; 1.60) and 1.15 (CI 0.89; 1.49), respectively]. While the authors did not observe an increased meta-RR for solvent exposure overall [meta-RR 1.00 (CI 0.73; 1.35)], a slight increase of oral cancers was observed with an exposure to organic solvents and chlorinated solvents.

Tarvainen et al. (38) found a significantly increased incidence of oral cavity and pharyngeal cancer (analyzed together) in finish workers exposed to high cumulative levels of hydrocarbon solvents [SIR 1.97 (CI 1.23; 2.98)] and a borderline significant risk for high cumulative exposure to aromatic hydrocarbons [SIR 1.50 (CI 0.99; 2.19)]. Coble et al. (39) reported an exposure–response trend for cumulative exposure to solvents in men, with an OR reaching 3.2 (CI 0.8;12.6; p for trend = 0.03) in the highest exposure category. Barul et al. (40) found no association between chlorinated solvent exposure and head and neck cancer risk, although they observed a non-significant increased risk among subjects who had the highest cumulative perchloroethylene exposure levels [OR 1.81 (CI 0.68; 4.82)] and methylene chloride [OR 1.42 (CI 0.70; 2.87)]. They further found a borderline significant increased risk of oral cancer for exposure to tetrahydrofuran [OR 1.87 (CI 0.97; 3.61)], no exposure-response trend was observed.

Moreover, Carton et al. (41) in a case–control study of 296 head and neck SCC in women (including 88 oral cavity cancers), and 775 women controls, reported an association of oral cavity cancers with trichlorethylene exposure [OR 2.12 (CI 0.97; 4.60)], with a dose–response relationship [OR 6.84 (CI 2.11; 22.1) for exposure duration >10 years; OR 2.73 (CI 1.02; 7.30) for a cumulative exposure index >median]. The authors further reported a significantly increased risk of oral cavity cancer among women exposed for more >10 years to white spirits [OR 2.51 (CI 1.25; 5.02)]. For benzene, no epidemiological evidence for an association with oral cavity cancers was identified. Of note, the IARC monograph on the carcinogenicity of benzene reported a significantly increased incidence of squamous cell carcinoma of the oral cavity in male and female rats in two oral gavage studies, with exposure pattern (i.e., daily administration as a single bolus over a longer period), close to the patient’s exposure condition (42).

Overall, current available evidence supports the hypothesis of an increased risk of oral cancer for exposure to aromatic hydrocarbons, organic and chlorinated solvents. While the results for an association with printing processes are diverging, most studies report an elevated, sometimes significant risk. Some limitations have to be considered in interpreting the current evidence. No study individually assessed the risk in gingiva SCC, and several studies grouped oral cavity and pharyngeal cancers. While these localizations share major risk factors (i.e., smoking and alcohol consumption), they also present some etiologic heterogeneity, in particular regarding HPV, a risk factor of growing importance for oropharyngeal cancer. Information on HPV infection was generally not available in the studies reviewed. Moreover, a limitation frequently observed in epidemiological studies investigating occupational exposure, is the difficulty to consider individual variation on exposures within the same job title, which may generate non-differential misclassification of exposure, and result in bias toward the null or alter dose–response trends (43). In particular, none of the identified studies considered hazardous work practices using mouth for solvent siphoning. Also, there was a lack of information about the cancerogenic consequences of oral siphoning solvents, and the underlying molecular mechanisms linking solvent exposure to oral cavity cancers remain poorly understood. Of note, several studies reported an increased risk of cytogenetic damage in oral mucosal epithelial cells in petrol station attendants and petrol pump workers (44–46), as well as in painters (47) and construction workers (48) exposed to petrol products, compared to unexposed controls. Petrol is a complex combination of hydrocarbons; mainly aliphatic and alicyclic compounds and to a lesser extent aromatic compounds predominantly benzene, toluene, and xylene. Owing to the influence of geno- and cytotoxicity on chemical carcinogenesis, these observations are of interest for the present case report.

## Conclusion

In absence of major extraprofessional exposures in this 56-year-old never smoker patient with low alcohol consumption, this case report and review of the literature contributes to prompt the hypothesis of an association of the HPV negative gingiva SCC with regular occupational oral solvent exposure through mouth siphoning. While the available evidence remains limited to formally establish a causal relationship, the literature supports a positive association of SCC of the upper aero digestive tract with occupational exposures to solvents and printing processes. Given the frequency of OSCC, it is important to raise awareness among head and neck, and oral medicine specialists to investigate occupational exposures in their patients. In particular the hazardous work practice of oral siphoning the present patient is likely to be underestimated, suggesting that clinicians should investigate these practices in patients with occupational history of solvent use. Moreover, this case report stresses the need for further epidemiological and mechanistic research to better understand the role of occupational solvent exposures, in the development of OSCC, including types of solvents, modes of exposure, as well as relation with specific OSCC subsites and molecular profiles.

## Patient perspective

“The lesion was first seen and managed by my dentist, who referred me to the Cancer Center when it recurred. At the time of diagnosis, I did not make a connection with the exposures at work. It was the head and neck surgeon at CLB who first asked me about my occupational history and exposures and further referred me to the occupational cancer consultation.

In my position as screen printer, I have worked in a small enterprise, and I was the only screen printer. For economic reasons, solvents were ordered in large drums, but there were no instruments to transfer the solvents into smaller containers. You had to make it with what you had, and there was no information on the dangerousness of products nor the priming the siphon tube using mouth suction. It is almost unavoidable to get it in your mouth, and sometimes you swallow it by accident. To clean the screen, I had to lie under it while cleaning it with solvent-soaked cloths. It was very irritating for the eyes, which burned regularly. There was no personal protective equipment in this position.

I wanted to stress the importance of preventive procedures in small businesses, which do not always have the resources.”

## Data availability statement

The raw data supporting the conclusions of this article will be made available by the authors, without undue reservation.

## Ethics statement

Written informed consent was obtained from the individual(s) for the publication of any potentially identifiable images or data included in this article.

## Author contributions

CL: Writing – original draft. LB: Investigation, Writing – review & editing. PZ: Supervision, Validation, Writing – review & editing. BC: Supervision, Validation, Writing – review & editing. BF: Conceptualization, Data curation, Supervision, Validation, Writing – original draft, Writing – review & editing.
